# Exhaustive expansion: A novel technique for analyzing complex data generated by higher-order polychromatic flow cytometry experiments

**DOI:** 10.1186/1479-5876-8-106

**Published:** 2010-10-30

**Authors:** Janet C Siebert, Lian Wang, Daniel P Haley, Ann Romer, Bo Zheng, Wes Munsil, Kenton W Gregory, Edwin B Walker

**Affiliations:** 1 CytoAnalytics, Denver, CO, USA; 2 Oregon Medical Laser Center, Providence St. Vincent Medical Center, Portland, OR, USA; 3 Robert W Franz Cancer Research Center, Earle A. Chiles Research Institute, Providence Cancer Center, Portland, OR, USA

## Abstract

**Background:**

The complex data sets generated by higher-order polychromatic flow cytometry experiments are a challenge to analyze. Here we describe Exhaustive Expansion, a data analysis approach for deriving hundreds to thousands of cell phenotypes from raw data, and for interrogating these phenotypes to identify populations of biological interest given the experimental context.

**Methods:**

We apply this approach to two studies, illustrating its broad applicability. The first examines the longitudinal changes in circulating human memory T cell populations within individual patients in response to a melanoma peptide (gp100_209-2M_) cancer vaccine, using 5 monoclonal antibodies (mAbs) to delineate subpopulations of viable, gp100-specific, CD8+ T cells. The second study measures the mobilization of stem cells in porcine bone marrow that may be associated with wound healing, and uses 5 different staining panels consisting of 8 mAbs each.

**Results:**

In the first study, our analysis suggests that the cell surface markers CD45RA, CD27 and CD28, commonly used in historical lower order (2-4 color) flow cytometry analysis to distinguish memory from naïve and effector T cells, may not be obligate parameters in defining central memory T cells (T_CM_). In the second study, we identify novel phenotypes such as CD29+CD31+CD56+CXCR4+CD90+Sca1-CD44+, which may characterize progenitor cells that are significantly increased in wounded animals as compared to controls.

**Conclusions:**

Taken together, these results demonstrate that Exhaustive Expansion supports thorough interrogation of complex higher-order flow cytometry data sets and aids in the identification of potentially clinically relevant findings.

## Background

Flow cytometry (FCM) is a powerful technology with major scientific and public health relevance. FCM can be used to collect multiple simultaneous light scatter and antigen specific fluorescence measurements on cells as each cell is excited by multiple lasers and emitted fluorescence signals are passed along an array of detectors. This technology permits characterization of various cell subpopulations in complex mixtures of cells. Using new higher-order multiparameter FCM techniques we can simultaneously identify T and B cell subsets, stem cells, and specific cell surface antigens, cytokines, chemokines, and phosphorylated proteins produced by these cells. Higher order FCM allows us to measure at least 17 parameters per cell [1], at rates as high as 20,000-50,000 cells per second.

Increasing sophistication in FCM, coupled with the inherent complex dimensionality of clinical and translational experiments, leads to data analysis bottlenecks. While the literature documents a long history of automated approaches to gating events within a single sample [2-4], the gated data remains complex, with readouts for tens to hundreds of phenotypes per sample, multiple samples per patient, and multiple cohorts per study. Unfortunately, there is a paucity of proven analytical approaches that provide meaningful biological insight in the face of such complex data sets.

Furthermore, interpretation of results from higher order experiments may be biased by historical results from simpler lower order experiments. Marincola [5] suggests that modern high-throughput tools, coupled with high-throughput analysis, provide a more unbiased opportunity to reevaluate the basis of human disease, while advocates of cytomics [6,7] observe that exhaustive bioinformatics data extraction avoids the inadvertent loss of information associated with *a priori *hypotheses. Fundamentally, these authors underscore the distinction between inductive (hypothesis-generating) and deductive (hypothesis-driven) reasoning. This distinction is clearly applicable to the interpretation of higher-order multiparameter flow cytometry data. Herein, we apply a powerful inductive data analysis approach to two distinctly different studies in order to demonstrate its broad applicability. The first study examines human memory T cell responses to a melanoma peptide cancer vaccine, while the second inspects porcine stem cell phenotypes associated with wound healing.

In a previously described melanoma booster vaccine study [8], we used 8-color FCM to characterize the phenotypes of viable (7AAD^-^) melanoma antigen-specific (gp100 tetramer^+^) CD8^+ ^T cells collected from peripheral blood. Memory and effector T cell subpopulations responding to vaccine antigen were characterized using 5 additional monoclonal antibodies (mAbs) specific for CCR7, CD45RA, CD57, CD27, and CD28. Samples were collected from 7 donors at 3 time points: after (post) the initial vaccine regimen (PIVR); at a long term memory (LTM) time point collected 18 to 24 months after the end of vaccine administration; and after two boosting vaccines (P2B). Phenotypes for T_CM _have been described based on lower-order 3-4 color staining with different combinations of the above antibodies, with data suggesting a consensus T_CM _phenotype of CCR7+CD45RA-CD57-CD27+CD28+. We demonstrated that LTM gp100-specific CD8^+ ^T cells were enriched for this consensus phenotype [8]. We also described a gp100-specific T_CM _subset that retained CD45RA expression (CCR7+CD45RA+CD57-CD27+CD28+), which we termed T_CMRA, _and which may represent a T_CM _precursor population similar to that described in the mouse [9]. Although this consensus phenotype has previously been used to primarily define naïve T cells, it clearly characterized a subpopulation of antigen-educated (i.e. gp100 tetramer positive) long term memory CD8^+ ^T cells in the melanoma vaccine study. This phenotype signature may delineate a T_CM _precursor population that arises shortly after antigen activation of naïve T cells. Thus, studies in the mouse demonstrate that tumor-specific T_CM _and similar putative T_CM _precursors, referred to as central memory stem cells (T_SCM_), which may derive from early daughter cell division after antigen stimulation of naïve T cells, express elevated levels of proliferation, enhanced survival *in vivo*, and superior CTL function compared to effector or effector-memory (T_EM_) T cells [9]. However, the origin of T_CM _and T_SCM _precursors remains controversial, since other data supports the hypotheses that such memory subpopulations may also develop from effector and effector-memory T cells [10]. Controversy aside, enhanced proliferative and survival properties characteristic of memory T cells have been correlated with anti-tumor responses in mice and humans receiving adoptive T cell-based therapies [11]. Thus, the use of higher-order flow cytometry and comprehensive multiparameter data analysis could facilitate the identification and expansion of T_CM _and T_CM _precursor subpopulations (i.e. T_SCM_) for more effective cancer immunotherapy regimens. However, such a therapeutic strategy would depend on first demonstrating memory T cell functional properties by sorted cells exhibiting such putative memory phenotype signatures.

Our second study examines complex stem cell phenotypes mobilized in response to wound healing. One use of stem cell therapy may be that of repairing damaged tissues, since bone marrow stem and progenitor cells can differentiate into muscle cells, endothelial cells, and nerve cells *in vitro *and *in vivo *[12]. Extremity injuries complicated by compartment syndrome (e.g. trauma-related severe swelling that can lead to ischemia and permanent tissue necrosis) are a common consequence of battlefield trauma, crush injuries that have been reported in recent earthquakes, and many sport injuries. While faciotomy can reduce the injury, there is no treatment that replaces or regenerates muscle and nerve tissues, leaving the patient with a permanent disability [13]. Human studies have demonstrated that injection of bone marrow stem cells into ischemic muscle may reduce the damage to the muscle and the loss of muscle function [14-18]. We have hypothesized that healthy, autologous bone marrow stem cells could be used to treat compartment syndrome. Our initial investigation focused on determining the optimal time to harvest bone marrow stem and progenitor cells after injury in the event that injury might amplify the mobilization of stem cell populations in the bone marrow. Bone marrow samples were collected from 8 injured swine and 8 control swine at pre-injury (baseline) and at 4 consecutive one-week intervals. Bone marrow was characterized by 5 different staining panels consisting of 8 mAbs each, as presented in Table 1. In total, 12 different monoclonal antibodies (CD29, ckit, CD56, CXCR4, CD105, CD90, Sca-1, CD44, CD31, CD144, CD146, and VEGFR2) were used. Others have used more restrictive lower order combinations of these markers to delineate mesenchymal stem cells (CD29, CD90, and CD44) [19,20], primitive stem cells (ckit, CXCR4, and Sca-1)[21-23], myoblasts (CD56 and CXCR4) [24,25], and vascular-relative cells (CD146, CD31, CD144, CD105, and VEGFR2) [26-29]. However, to date, there has been no description of the combined use of all of these putative progenitor cell set descriptors in higher order staining panels.

**Table 1:** Five monoclonal antibody panels for stem cell study.

Panel	Main	CD31	CD144	CD146	VEGFR2
Antibody	CD29	CD29	CD29	(CD146)	CD29

	ckit	(CD31)	(CD144)	ckit	ckit

	CD56	CD56	CD56	CD56	CD56

	CXCR4	CXCR4	CXCR4	CXCR4	CXCR4

	CD105	CD105	CD105	CD105	CD105

	CD90	CD90	CD90	CD90	(VEGFR2)

	Sca-1	Sca-1	Sca-1	Sca-1	Sca-1

	CD44	CD44	CD44	CD44	CD44

Each of the 5 panels consists of 8 mAbs. The differences from the main panel are indicated both in the name of the panel and by the antibody listed in parentheses.

Our multiparameter studies allow the identification of hundreds to thousands of phenotypes of cells, based on combinations of positive or negative expression of the included mAbs. For example, in the melanoma vaccine study, we initially considered all 32 (2^5^) possible phenotypes defined by positive and negative combinations of all 5 variable markers, e.g. CCR7+CD45-CD57-CD27+CD28+ [8]. This type of analytical strategy is used by many researchers [30-32]. However, it focuses on populations defined by exactly the number of variable parameters in the staining panel (5, in the case of the vaccine study). Thus, to more thoroughly explore the data, we exhaustively expanded the data sets to include all possible phenotypes defined by combinations of 0, 1, 2, 3, 4, and 5 markers, e.g. CCR7+ and CCR7+CD57-CD27+CD28+. When each marker can assume one of two values (positive or negative), the number of possible cell subsets in an M-marker study is 2^M^. When each marker can assume one of three possible values (positive, negative, or unspecified), the number of possible cell sets is 3^M^, or 3^5 ^(243) in this 5 marker study, as illustrated in Table 2. In the wound healing study, bone marrow was characterized by 5 different 8 color panels. Exhaustive Expansion of these 8 marker sets to include all possible 0, 1, 2,...8 marker sets resulted in 6,561 (3^8^) sets per panel, for a total of 32,805 (6,561 × 5 panels) cell subpopulations per sample.

**Table 2:** Combinations of positive/negative phenotypes in a 5-marker panel.

Number of markers (M)	Number of +/- gates given M markers (G)	Combinations	Number of combinations of M markers in a 5 marker panel (C)	Number of gates times number of combinations (G × C)
0	2^0 ^= 1	No markers specified	1	1

1	2^1 ^= 2	A, B, C, D, E	5	10

2	2^2 ^= 4	AB, AC, AD, AE, BC, BD, BE, CD, CE, DE	10	40

3	2^3 ^= 8	ABC, ABD, ABE, ACD, ACE, ADE, BCD, BCE, BDE, CDE	10	80

4	2^4 ^= 16	ABCD, ABCE, ABDE, ACDE, BCDE	5	80

5	2^5 ^= 32	ABCDE	1	32

				TOTAL = 243

This table illustrates the total number of positive/negative gates in a 5-marker panel, with hypothetical markers A, B, C, D and E. There are five possible 1-marker combinations, ten 2-marker combinations, ten 3-marker combinations, five 4-marker combinations, and one 5-marker combination. For each combination, there are 2^M ^positive/negative gates where M is the number of markers in the combinations. Thus, there are 243 possible phenotypes in a 5 marker experiment. This generalizes to 3^M^.

Since we could not manually analyze data from hundreds to thousands of phenotypes efficiently, we first identified numerically interesting phenotypes by computing metrics for all derived sets. For example, in the melanoma vaccine study, the middle of three time points represented a long term memory time point, collected 18 to 24 months after exposure to the vaccine antigen. Consequently, one feature of interest was the delineation of phenotypes that peaked at this long term memory time point. In the wound healing study, since there were both wounded animals and control animals, we could identify phenotypes in which the expression levels for the wounded animals were greater than the levels for the control animals. In each case, simple visualizations, such as those presented in the Results, illustrated the patterns of response and helped us vet the numerically interesting phenotypes for biological relevance. In both studies we identified results with possible important clinical implications that would have been very difficult to find using standard analytical techniques. Using Exhaustive Expansion we were able to define a putative minimum obligate phenotype for central memory T cells, and delineate multiple bone-marrow-derived putative myogenic MSC subpopulations that may be mobilized in response to myonecrotic injury.

## Methods

### Melanoma Vaccine Study

The clinical trial protocol and the flow cytometry staining and analysis procedures used to acquire data in this study have been described in detail elsewhere [8,33]. Briefly, early stage melanoma patients were vaccinated every second or every third week over six months with a modified, HLA-A2 restricted melanoma associated peptide, gp100_209-2M_. Leukophereses were collected before the vaccine regimen, after (post) the initial vaccine regimen (PIVR); at a long term memory (LTM) time point 18-24 months later; and following two additional boosting vaccines (P2B) given at one month intervals following the LTM leukopak collection. The protocol was reviewed by NCI's CTEP and approved by the Providence Health System institutional review board. All patients gave written informed consent. Cryopreserved PBMCs from PIVR, LTM and P2B time points were stained simultaneously with gp100 tetramers and with mAbs specific for CD8β, CCR7, CD45RA, CD57, CD27, CD28, and with 7AAD to discriminate live from dead cells. All samples were analyzed on a 9 color Beckman Cyan ADP flow cytometer. Viable lymphocytes were gated for positive CD8β and gp100 tetramer staining, and gp100-specific CD8β^+ ^T cells were further interrogated for expression of the remaining five cell surface markers (CCR7, CD45RA, CD57, CD27, and CD28) to determine their subphenotypes. At least 5,000 gp100-specific CD8β^+ ^T cells were collected per sample. All data was acquired in FCS format (Summit 4.2) and analyzed using the FCOM format of Winlist 5.0 Software (Verity House Software). "Fluorescence minus one" (FMO) controls were used to define positive and negative histogram staining regions for each fluorescent variable.

### Porcine Stem Cell Study

All protocols were approved by the IACUC of Legacy Research and Technology Center. A bilateral compartment syndrome injury was produced in the anterior tibialis muscles by infusing porcine plasma directly into the muscles. A standardized bone marrow collection procedure was used as previously described [34], with bone marrow harvested from the tibia of anesthetized swine. Bone marrow was transferred to an automated cell processing system, BioSafe SEPAX cell separating system (Biosafe SA, Bern, Switzerland), within 60 minutes of collection, and mononuclear cells were isolated. Each sample was divided into 5 aliquots, which were stained for surface marker expression as summarized in Table 1. All samples were acquired using a BD™ LSR II flow cytometer.

To identify ckit (a.k.a stem cell factor (SCF)) expression, a porcine SCF ligand conjugated with biotin, kindly provided by Dr. Christene Huang (Transplantation Biology Research Center at Massachusetts General Hospital), was used together with a streptavidin-PE (Jackson Immunoresearch, West Grove, PA) for secondary binding. The antibodies for the other markers were all commercial monoclonal antibodies which were specific for porcine antigens or were anti-human or anti-mouse which cross react with the designated epitopes in swine: CD29-FITC, CD146-FITC and CD105 (GeneTex Inc., Irvine, CA), CD90-APC and CD44-APC-Cy7 (BioLegend, San Diego, CA), CD56-PE-TR (Invitrogen, Carlsbad, CA), Sca-1-Alexa Fluor 700 (Sca-1-AF700), CXCR4-PE-Cy7 (eBioscience, San Diego, CA), CD31-PE (AbD Serotec, Raleigh, NC), CD144-PE (Santa Cruz Biotechnology, Santa Cruz, CA), and VEGFR2-APC (R&D Systems, Minneapolis, MN). The anti-CD105 antibody was conjugated with Pacific Blue using a monoclonal antibody labeling kit (Invitrogen, Carlsbad, CA), following manufacturer's protocol.

### Systems and Software

**Figure 1:**
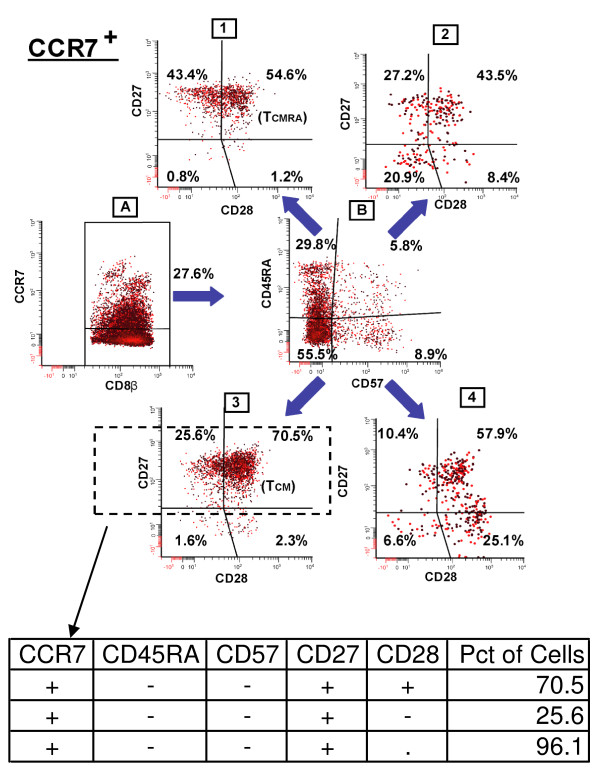
**Representative gating strategy and additional phenotype set calculations**. This figure illustrates a gating strategy in which CCR7^+ ^cells are further categorized by positive or negative expression of CD45RA and CD57. Cells in each resulting quadrant (dot plot B) are then categorized based on CD27 and CD28 staining frequencies (dot plots 1-4). The callout table illustrates how the two phenotypes CCR7+CD45RA-CD57-CD27+CD28+ (+--++) and CCR7+CD45RA-CD57-CD27+CD28- (+--+-), marked by dotted lines, are aggregated to form a superset population, CCR7+CD45RA-CD57-CD27+ (+--+.), in which CD28 expression is unspecified.

While the details of the data analysis approach are provided in the Results, we highlight the system components below. The "Expander" program for deriving all possible phenotypes or sets is implemented in the Java programming language, and is freely available upon request. Input consists of a comma-delimited file containing fields for absolute set or phenotype names, 3 additional qualifiers, and the percentage of cells in the set specified by the name and the qualifiers. Output consists of a comma-delimited file containing fields for 3 qualifiers, the relative set name, and the derived data value. The three qualifiers from the input are passed to corresponding rows in the output without modification. These qualifiers support downstream analysis based on characteristics such as donor, time point, and treatment protocol. Representative input and output formats are shown in Table 3. Relative set names and their derivation are illustrated in Figure 1 and described in the associated results. The derived data values are simply the sum of the frequencies of the relevant subsets. The output was then loaded into a relational database (MySQL), and standard SQL statements and graphing utilities were used to interrogate the data. Statistical tests were performed using the R software environment for statistical computing (http://www.r-project.org).

**Table 3:** Representative input and output for the "Expander" program.

Representative Input
CCR7+CD45+CD57-CD27+CD28-, panel, EA02, LTM,2.48

CCR7+CD45+CD57-CD27+CD28+, panel, EA02, LTM,5.41

CCR7+CD45+CD57+CD27-CD28-, panel, EA02, LTM,1.47

CCR7+CD45+CD57+CD27-CD28+, panel, EA02, LTM,0.22

CCR7+CD45+CD57+CD27+CD28-, panel, EA02, LTM,0.34

CCR7+CD45+CD57+CD27+CD28+, panel, EA02, LTM,1.34

**Representative Output**

panel, EA02, LTM,+++++,1.34

panel, EA02, LTM,++++-,0.34

panel, EA02, LTM,++++.,1.68

panel, EA02, LTM,+++-+,0.22

panel, EA02, LTM,+++--,1.47

panel, EA02, LTM,+++-.,1.69

panel, EA02, LTM,+++.+,1.56

panel, EA02, LTM,+++.-,1.81

The Expander program derives aggregate sets or supersets from input data, and outputs both the relative set name and the percentage of cells in both the newly derived sets and the original sets. The percentage of cells in the derived sets is calculated by adding together the percentages in the subsets, as illustrated in Figure 1. The rows below illustrate the format of both input and output, but not direct correspondence between input and output. Output is loaded into a relational database for further analysis.

### Statistical Methods

In the melanoma vaccine study, the Wilcoxon signed-rank test was used to identify either increased expression between time points or decreased expression between time points, depending on the pair of time points under consideration. The p-values were then used to screen populations for biologically meaningful results. These p-values provided a simple, well-understood metric to encapsulate the differences between the two time points. An alternative metric, such as 4 of 7 donors showing at least a 5% change between time points, would have been more verbose and would have required more detailed justification. In the porcine wound healing study, the Wilcoxon rank sum test was used to identify phenotypes in which the wounded cohort showed a greater change from baseline than did the control cohort.

## Results

### Exhaustive Expansion

In both studies, standard FCM analysis software was used to establish positive and negative gates based on the use of "fluorescence-minus-one" (FMO) controls for the included markers. In the case of the 5 memory markers used in the melanoma vaccine study, 32 (2^5^) sets were subsequently generated using WinList's™ (http://www.vsh.com) FCOM function. Such combination gates also can be generated with other flow cytometry analytical software such as FlowJo (http://www.flowjo.com) and FCS Express (http://www.denovosoftware.com). The gating strategy for this study is illustrated in Figure 1. By inspecting a series of two-dimensional scatter plots, positive and negative gating boundaries were set, dividing the cells into subpopulations. Each of the 4 quadrants in dot plots 1 through 4 illustrates the frequencies of phenotypes of gp100 tetramer^+ ^CD8^+ ^T cells that are defined by positive and negative combinations of CCR7, CD45RA, CD57, CD27, and CD28.

Next we derived the percentage of cells in the more comprehensive analysis of all 243 (3^5^) possible phenotypes, as defined by 0, 1, 2,... 5 parameters, using a custom Java program as described in the Methods. We utilize a shorthand notation for phenotypes by introducing a placeholder (".") to represent an unspecified parameter. These concepts are also illustrated in Figure 1, in which the callout table shows the shorthand notation for 2 populations specified by 5 markers, CCR7+CD45RA-CD57-CD27+CD28+ (+--++) and CCR7+CD45RA-CD57-CD27+CD28- (+--+-). The table also shows the notation for the 4 marker phenotype (+--+.) resulting from the summation of the frequencies of the two 5 marker phenotypes. Notice that CD28 assumes 3 values, "+", "-", and ".". The phenotype +--+. represents the combination or union of two subphenotypes or subsets (+--++ and +--+-), Hereafter, subphenotype signatures will be referred to as either sets or phenotypes.

The universal set (.....) contains 100% of the cells in the population of interest (e.g. viable, antigen-positive, CD8^+ ^cells), and thus serves as an internal control. All other sets are proper subsets of the universal set. As presented here, Exhaustive Expansion applies to binary classification systems (e.g. positive and negative gating), but extension to n-ary classification systems (e.g. dim, intermediate, bright) is possible. After derivation of frequencies for all sets, data was loaded into a relational database (MySQL) and analyzed with SQL statements and graphing utilities.

### Melanoma Vaccine Study

#### Average CV Suggests Stable CD27, CD28, and CD45RA Expression Over Time

**Figure 2:**
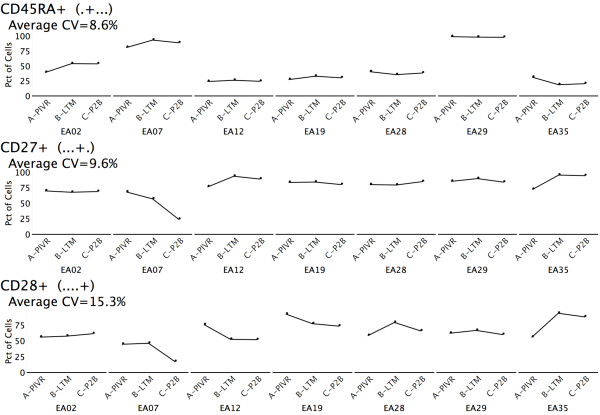
**Longitudinal single parameter frequency profiles for 7 patients across 3 time points**. Frequencies of CD45RA+, CD27+, and CD28+ gp100-specific CD8^+ ^T cells are shown for each patient (EA02, EA07...) for each of 3 time points (PIVR, LTM, P2B). The Average CV (CV computed for each patient, then all 7 patients averaged) is shown for each phenotype. All 3 Average CV values are less than 16%, suggesting stable expression over time for each of these cell surface parameters.

Having derived the percentage of cells in all 243 0- through 5-parameter sets in the melanoma vaccine study, we generated longitudinal profiles for all sets as shown by the example in Figure 2. This enabled us to clearly see the responses of each donor over time. Additionally, these profiles allow each donor to serve as his or her own control. Next, we looked for sets that were interesting based on coefficient of variation (CV, standard deviation divided by mean). We computed Average CV by calculating CVs for each donor across 3 time points, and then averaging the 7 CVs. We then sorted the longitudinal profiles both by ascending average CV and descending average CV. In this data, the sets with a low average CV, as shown in Figure 2, were particularly interesting because of their common use in lower order flow cytometry analysis to distinguish central memory and effector memory T cells [35,36]. At 8.59%, the CD45RA+ phenotype has the lowest Average CV of all 242 non-universal sets (those with at least one marker specified). In this case, even though there is inter-donor variation, the values are relatively stable over time for each individual donor. There are 4 donors with relatively low levels of CD45RA expression, 2 donors with relatively high levels, and 1 donor with an intermediate level. Thus, inspection reveals that the low Average CV was associated with donor stratification. Profiles for CD27+ and CD28+ are also shown in Figure 2, and similarly suggest overall low average CVs for individual patient phenotype frequencies over all 3 time points, but do not indicate inter-donor variation. Notably, all three of these markers are associated with the T_CM _consensus phenotype (CCR7+ CD45RA- CD57- CD27+ CD28+) predicted from lower order 3- and 4-marker flow cytometry analysis, yet individually show low to moderate frequency changes over the time course of the vaccine study, even though our previous data suggested T_CM _increased at LTM for most patients [8]. Since several studies have shown that early effector-memory T cells (T_EM_) are also CD45RA- CD27+ CD28+ [8,35,36], the stability in expression of each of these single markers over time may reflect the redistribution of gp100-specific memory CD8^+ ^T cells from the T_EM _to the T_CM _phenotype compartment at LTM. Conversely, by this line of reasoning, higher frequencies of memory T cells may be expected to be distributed in the T_EM _phenotype compartment after antigen challenge at PIVR and P2B.

#### Peak Finding Algorithm Highlights Central-Memory-Like Phenotype

Arguably, in situations of acute primary antigen challenge, such as the gp-100 vaccine regimen, central memory phenotypes (T_CM_) should be more predominant 18 to 24 months after antigen exposure, represented by a peak frequency at time point B (LTM). Both effector and early and late stage effector memory phenotypes should be more predominant after recent secondary antigen exposure, represented by an increase in these phenotypes (and a concomitant decrease in T_CM_) following boosting immunizations at time point C (P2B). Thus, to identify specific patterns of longitudinal changes, we computed p-values (Wilcoxon signed-rank test, a paired test) between pairs of time points for each phenotype.

To identify the T_CM _peaks, we looked for phenotypes that showed a statistically significant increase from A to B, and a concomitant decrease from B to C. Twenty three sets met these criteria with p-values less than 0.05. Eleven sets met these criteria with p-values less than 0.01. We inspected the longitudinal profiles for all 11 sets to verify the presence of reasonable peaks. We did not correct for multiple comparisons because we simply used the p-values as a numeric indicator of changes across the population, giving us direction for visual inspection. Furthermore, we did not make family-wide conclusions about the statistical significance of the peaks. We call the algorithm used in this analysis a "peak finding algorithm." A similar approach could be used to find valleys.

**Figure 3:**
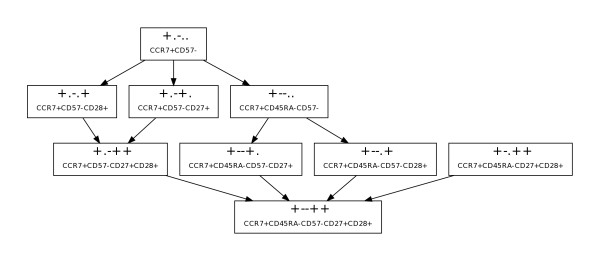
**Phenotype hierarchy of central-memory like sets**. The graph shows the family or hierarchy of 9 sets that match the criteria for long term memory peaks (statistically significant increases from time point A to time point B, and decreases from time point B to time point C, with P < 0.01 for each comparison), and are supersets or parent sets of the consensus central memory phenotype of CCR7+CD45RA-CD57-CD27+CD28+ (+--++).

**Figure 4:**
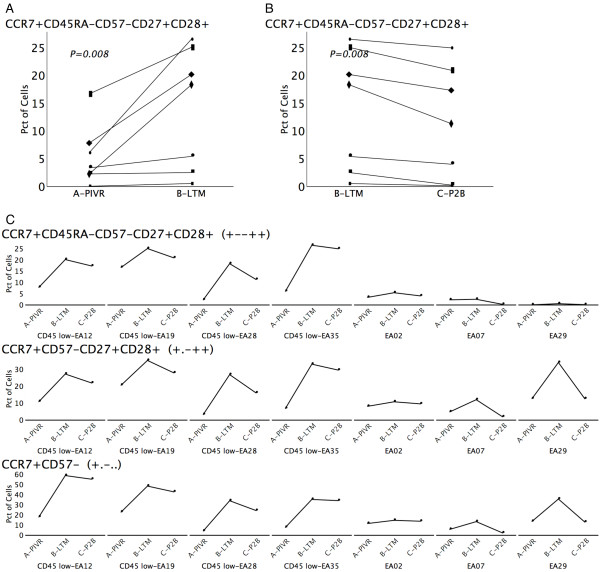
**Long-term frequency changes for the T_CM _consensus phenotype, CCR7+CD45RA-CD57-CD27+CD28+ (****+--++****) and two associated supersets**. (A) Plot illustrating the statistically significant increase in the T_CM _consensus phenotype frequency between PIVR and LTM for all 7 patients. (B) The concomitant decrease between LTM and P2B for the frequency of the consensus T_CM _phenotype. (C) The longitudinal expression profile for the T_CM _consensus phenotype showing LTM peaks for 4 of 7 patients; longitudinal profile for the CD45RA unspecified superset, CCR7+CD57-CD27+CD28+ (+.-++), showing LTM peaks for 6 of 7 patients; and longitudinal profile for the CD45RA, CD27, and CD28 unspecified superset, CCR7+CD57- (+.-..), also showing LTM peaks for 6 of 7 patients. Data suggests CD45RA, CD27, and CD28 may not be obligate descriptors for central memory T cells.

Eight of the 11 sets with p-values less than 0.01 were supersets of the consensus T_CM _phenotype CCR7+CD45RA-CD57-CD27+CD28+ (+--++). These sets and the relationships between them are illustrated in the directed acyclic graph (DAG) shown in Figure 3. Since we derived supersets of cells by combining sets, this set inclusion hierarchy provides a tool to visualize the relationships between these sets. The terminal node of the DAG is the consensus T_CM _phenotype of CCR7+CD45RA-CD57-CD27+CD28+ (+--++). Figures 4A, 4B, and 4C illustrate the behavior of this phenotype over time. Figure 4A illustrates the changes from time point A to B for all 7 donors, while Figure 4B illustrates the changes from B to C. Figure 4C shows the longitudinal profile for all donors. The 4 CD45RA+ "low" donors, identified in Figure 2, exhibited correspondingly similar higher frequencies of the consensus T_CM _phenotype at time point B (LTM), and are shown on the left side of Figure 4C.

One of the phenotypes identified by the peak-finding algorithm was CCR7+CD57-CD27+CD28+ (+.-++), in which CD45RA is unspecified, and therefore includes both the CD45RA+ putative T_CM _precursor phenotype (T_CMRA_) and the CD45RA- T_CM _phenotype. The longitudinal profile for this set is shown in Figure 4C, and shows that 6 of 7 patients clearly peak at time point B. If the basic assumption that circulating gp100 specific CD8^+ ^T cells which are maintained 1-2 years after initial antigen exposure are both T_CM _and T_CMRA _is correct, this data confirms that CD45RA staining may not be obligate in identifying all long term central memory T cell subpopulations. This interpretation is reinforced by the donor-level consistency in CD45RA expression over time as illustrated in Figure 2. Fundamentally, if 3 donors (e.g. EA02, EA07, EA29) have relatively consistently high/intermediate frequencies of CD45RA staining over time, they are unlikely to show a peak in the 5-marker consensus phenotype characterized by negative expression of CD45RA at the LTM time point when frequencies of central memory subpopulations should be elevated. Similarly, CD27+ and CD28+ staining may not be obligate descriptors for T_CM_/T_CMRA _subpopulations since staining frequencies for both remain relatively stable (low average CVs - Figure 2) over time, and may simply reflect memory T-cell redistribution between T_EM _and T_CM_/T_CMRA _phenotype compartments. Concomitant CCR7+CD57- staining may prove to be a more definitive minimal obligate phenotype signature for T_CM_/T_CMRA _subpopulations. This is suggested by the observations that 6 of 7 patients show CCR7+CD57- peaks at LTM (Figure 4C), and that 7 of the 9 sets in Figure 3 are subsets of the CCR7+CD57- (+.-..) phenotype.

### Porcine Stem Cell Study

#### Screening of Thousands of Subpopulations Identifies Novel Stem Cell Phenotype

In the porcine wound-healing study, Exhaustive Expansion was applied to 5 different 8-parameter data sets generated using WinList's FCOM function, after setting positive and negative staining regions for each marker with FMO controls. This resulted in delineation of 6,561 (3^8^) sets per sample per panel. Next, we computed changes from baseline (e.g. week 1 results minus week 0 results) for all phenotypes for all donors for weeks 1 through 4. We did not see clear kinetic changes in this data over the 4 week period, perhaps because these changes occurred much earlier, during the interval between week 0 and week 1, when no samples were drawn. Thus, to look for changes from baseline across the time frame of the study, we averaged the change from baseline data for each donor for each cell population over the 4 observations made in week 1 through week 4. Hereafter, we refer to this metric as the average delta value.

Additionally, we defined a process control range, based on analysis of 6 aliquots from a single animal drawn at a single point in time. For each phenotype, the process control range was defined as the maximum frequency value of the 6 replicates minus the minimum frequency value. This provided a conservative approach to quantifying the precision of our assay, and allowed us to focus on phenotypes with readouts exceeding the process control range.

**Figure 5:**
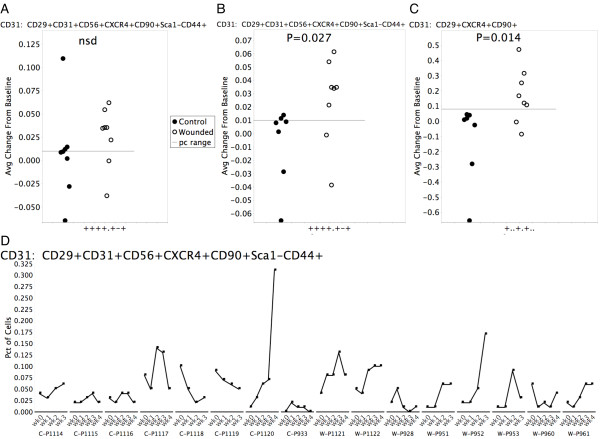
**Differences between control and wounded animals for 2 phenotypes from the CD31 panel**. (A) Average frequency change from baseline (average of frequency differences for week 1 minus week 0, week 2 minus week 0, week 3 minus week 0, and week 4 minus week 0) is shown for control animals (solid circles) versus wounded animals (open circles) for phenotype CD29+CD31+CD56+CXCR4+CD90+Sca1-CD44+ (++++.+-+). The horizontal line represents the process control range (maximum frequency minus minimum frequency, calculated from 6 replicate samples) for this phenotype. There is no significant difference between the cohorts, due in part to the outlier at approximately 0.115 for one animal in the control cohort. (B) The same phenotype analysis with outlier removed shows a statistically significant difference between wounded and control cohorts. (C) Frequency differences between wounded and control animals for the phenotype superset, CD29+CXCR4+CD90+ (+..+.+..), which was common to 19 of the putative myogenic precursor phenotypes shown in Table 4. (D) Longitudinal profiles for all animals for week 0 through week 4 for set CD29+CD31+CD56+CXCR4+CD90+Sca1-CD44+ (++++.+-+). Control animals indicated by C, Wounded by W. Note the week 4 outlier on control animal C-P1120. This animal was removed from the analysis shown in (B) and (C).

Next, to identify populations of numeric interest, we identified sets in which 6 or more (out of 8) wounded animals had an average delta greater than the process control range, and 6 or more control animals had an average delta less than or equal to the process control range. The resulting 122 sets (0.4% of the total 32,805 sets) came from three of the five panels, with two panels having no sets that matched these criteria. Of the 122 sets, 76 had p-values (Wilcoxon rank sum, one-sided) less than 0.05. Twenty-three of these 122 phenotypes were positive for CD29 (β1-integrin) and CXCR4, which are indicative of muscle progenitor cells in mouse models [25,37]. All of these CD29+CXCR4+ sets were from the CD31 panel. Initially, none of these sets showed statistically significant differences between wounded and control populations, due at least in part to the presence of an outlier in the control group, as shown by the scatter plots in Figure 5A. This outlier was driven by an unusually large observation for one of the donors, which in the case of the CD29+CD31+CD56+CXCR4+CD90+Sca1-CD44+ (++++.+-+) phenotype was an extreme outlier (greater than quartile 3 plus 3 times the interquartile range), and nearly twice as large as the next largest observation (.31% versus .17%). This outlier observation from week 4 for control animal C-P1120 is illustrated in Figure 5D. When this animal was removed from the analysis, all 23 of the CD29+CXCR4+ phenotypes showed statistically significant differences between the control and wounded animals. Two of these phenotypes are shown in Figures 5B and 5C. Figure 5B shows the same phenotype as Figure 5A, only with the outlier removed. As the scatter plot shows one point per donor it better illustrates the details of the data than does a bar plot or box plot. Additionally, Figures 5A, B, and 5C have a reference line indicating the process control range. The 23 CD29+CXCR4+ phenotypes, itemized in Table 3, may represent different bone-marrow-derived mesenchymal progenitor cell populations mobilized in response to myonecrotic injury and capable of endothelial, chondrogenic, and myogenic differentiation. Notably, the superset CD29+CXCR4+CD90+ (Figure 5C) is common to 19 of the phenotypes in Table 4. As such it may indicate a minimum obligate progenitor cell phenotype.

**Table 4:** 23 CD29+CXCR4+ subsets showing significant differences between wounded and control animals.

Panel	Relative Set Name	Absolute Set Name	P-Value
CD31	++++.+-+	CD29+CD31+CD56+CXCR4+CD90+Sca1-CD44+	0.027

CD31	++++.+-.	CD29+CD31+CD56+CXCR4+CD90+Sca1-	0.027

CD31	++.+-+-+	CD29+CD31+CXCR4+CD105-CD90+Sca1-CD44+	0.036

CD31	++.+-+-.	CD29+CD31+CXCR4+CD105-CD90+Sca1-	0.036

CD31	++.+-.-+	CD29+CD31+CXCR4+CD105-Sca1-CD44+	0.027

CD31	++.+-.-.	CD29+CD31+CXCR4+CD105-Sca1-	0.028

CD31	++.+.+-+	CD29+CD31+CXCR4+CD90+Sca1-CD44+	0.027

CD31	++.+.+-.	CD29+CD31+CXCR4+CD90+Sca1-	0.027

CD31	++.+.+.+	CD29+CD31+CXCR4+CD90+CD44+	0.02

CD31	++.+.+..	CD29+CD31+CXCR4+CD90+	0.02

CD31	+-++---.	CD29+CD31-CD56+CXCR4+CD105-CD90-Sca1-	0.027

CD31	+.++-+-+	CD29+CD56+CXCR4+CD105-CD90+Sca1-CD44+	0.02

CD31	+.++-+-.	CD29+CD56+CXCR4+CD105-CD90+Sca1-	0.02

CD31	+.++-+.+	CD29+CD56+CXCR4+CD105-CD90+CD44+	0.02

CD31	+.++-+..	CD29+CD56+CXCR4+CD105-CD90+	0.02

CD31	+.++.+-+	CD29+CD56+CXCR4+CD90+Sca1-CD44+	0.02

CD31	+.++.+-.	CD29+CD56+CXCR4+CD90+Sca1-	0.02

CD31	+.++.+.+	CD29+CD56+CXCR4+CD90+CD44+	0.02

CD31	+.++.+..	CD29+CD56+CXCR4+CD90+	0.02

CD31	+..+-+.+	CD29+CXCR4+CD105-CD90+CD44+	0.014

CD31	+..+-+..	CD29+CXCR4+CD105-CD90+	0.014

CD31	+..+.+.+	CD29+CXCR4+CD90+CD44+	0.014

CD31	+..+.+..	CD29+CXCR4+CD90+	0.014

Relative set name, absolute set name, and p-value (Wilcoxon rank sum, one-sided) are shown. P-values are calculated excluding data for one outlier control animal. These are also sets in which at least 6 of 8 wounded animals show average delta readouts greater than the process control range.

## Discussion

Here we have applied Exhaustive Expansion to two very different translational studies to demonstrate its broad application and utility. In each analysis, we generated all possible cell sets for each sample. Then we identified interesting sets based on coefficients of variation and long term memory peaks in the melanoma vaccine study, and separation between test and control cohorts in the wound healing study.

Analysis of data from multiparameter flow cytometry experiments consists of two main activities with well defined separation of concerns. First, events are gated into cell sets of interest using either manual or automatic techniques. Second, summary statistics describing these sets of cells are analyzed to identify meaningful experimental results. Exhaustive Expansion touches on both of these activities. In the case where positive/negative boundaries can be established for multiple markers, our Expander logic allows us to define a large number of supersets by exhaustively combining constituent subsets. Next, we identify features of interest such as Average CV, peaks, and separation between control and test cohorts. Such numeric features can be sorted and filtered, and illustrated with simple graphs. Importantly, these features are calculated for all phenotypes, thereby allowing systematic and relatively unbiased interrogation of the data. Additionally, the use of powerful mature software tools such as Java, MySQL, and R provides us with the flexibility to pursue the data analysis as suggested by the data itself and the underlying science.

For example, while we used a statistical test to quantify peaks in the melanoma study, we could have defined peaks based on an average fold change between time points (e.g. greater than 3), or on a criteria such as at least 4 donors showing at least a 5 percentage point change between time points. Alternatively, we could identify all phenotypes with a larger change than that shown by a predicted consensus phenotype. Or if we were interested in rare events, we could select sets in which less than 2 cells at baseline expanded to more than 20 cells after treatment. When a filter identifies many sets, the filter can be made more stringent. Alternatively, filters can identify a specific number or percentage of sets, such as the 10 sets with the largest average fold changes between two time points. Additionally, sets can be sorted on numeric characteristics such as fold change, p-value, or Average CV. This allows us to inspect sets ranked from largest to smallest fold change, for example, and perhaps further refine a threshold criteria based on some meaningful feature in the data. All of these numeric thresholds can and should be adjusted based on experimental conditions, assay precision, and the biological questions under investigation.

Adoptive transfer of tumor specific T cells in cancer immunotherapy translational studies has previously emphasized the transfer of highly differentiated, end stage effector T cells from *in vitro *IL-2 supported expansion cultures. More recently, compelling data from mouse tumor models suggests that tumor specific T_CM _and very early T_CM _precursors, referred to as central memory stem cells (T_SCM_), express elevated proliferation potential, enhanced long term survival *in vivo*, and give rise to activated CTLs *in vivo *with superior cytolytic activity compared to effector memory (T_EM_) or effector (T_EFF_) T cells from *in vitro *expansion cultures [9]. Adoptive transfer immunotherapy strategies based on the *in vitro *expansion of T_CM _and T_SCM _subpopulations may offer significant clinical advantage in treating cancer patients if the human phenotype signatures for T_CM _and T_SCM _can be identified, and rapid efficient recovery procedures are developed to recover memory cells for subsequent *in vitro *expansion [38-40].

Previously, in a clinical study of long term tumor specific T cell memory function in melanoma patients, we elucidated the multiparameter phenotype of tumor specific T_CM _(CCR7+CD45RA-CD57-CD27+CD28+), and a second potentially early T_CM _precursor which we referred to as T_CMRA _(CCR7+CD45RA+CD57-CD27+CD28+) [8]. Gp100-specific T_CMRA _shares its phenotype with naïve CD8^+ ^T cells, and thus may be similar to the T_SCM _subset described in the mouse. Sorting strategies to select for these highly defined putative central memory populations could thus be implemented prior to cytokine-mediated *in vitro *expansion and adoptive transfer. However, recovery strategies based on a more simple minimal obligate phenotype signature would facilitate the more rapid, efficient recovery of larger numbers of cells using bulk techniques such as magnetic bead separation. Exhaustive Expansion identified a possible minimal obligate T_CM_/T_CMRA _phenotype (CCR7+CD57-: Figure 4) that was common to 7/8 of the CCR7+ CD45RA-CD57-CD27+CD28+ supersets that showed frequency peaks at LTM (Figure 3). This putative minimal obligate T_CM_/T_CMRA _phenotype signature may thus facilitate the recovery of T_CM_/T_CMRA _T cells, and cells from the intermediate stages of the T_CMRA _to T_CM _to T_EM _differentiation pathway represented by the other superset phenotypes in Figure 3. Clearly, additional experiments, including functional assays, are required to validate the hypothesis that CCR7+CD57- is a minimal obligate phenotype for T_CM_.

A second somewhat unexpected outcome of Exhaustive Expansion of the melanoma specific CD8^+ ^T cell memory response was the suggestion that the combined frequency of tumor-specific T cells which express either the T_CM _or T_EM _phenotypes may not change appreciably over the course of the primary antigen challenge, long term memory maintenance, and following boosting immunization. The frequencies of gp100 specific T cells expressing key individual identifiers for the resolution of T_CM _and early T_EM _cells, such as CD45RA, CD27 and CD28, did not change appreciably across all three time points in the study (Figure 2). This may be explained in part by the observation that T_CM _and T_EM _phenotypes share the CD45RA-CD27+CD28+ signature [8,35,36]. The expression stability for each individual marker may suggest that, although cells may transition between the T_CM _and T_EM _phenotype compartments due to homeostasis-driven or antigen-stimulated proliferation, the overall combined frequency of the T_CM _plus T_EM _memory T cell pool as a fraction of all antigen specific T cells remains relatively constant. Thus, absolute numbers of cells in each compartment, and even the ratio of the frequency of cells with each phenotype, can fluctuate; but the total combined memory T cell frequency (i.e. T_CM _+ T_EM_) may remain relatively stable after primary immunization. This observation has important implications for the optimal design of primary immunization strategies in both infectious disease and cancer vaccine settings.

In the stem cell study, 8 color staining panels that included mAbs previously employed in lower-order panels to delineate mesenchymal cells (CD29, CD90, and CD44), primitive pluripotent stem cells (ckit, CXCR4, and Sca-1), differentiated myoblasts (CD56 and CXCR4), and vascular-relative cells (CD146, CD31, CD144, CD105, and VEGFR2) were used to more comprehensively characterize significant changes in bone-morrow-derived putative mesenchymal progenitor cell populations following myonecrotic injury. Our data analysis technique allowed us to identify novel populations by focusing on phenotypes that showed both statistically significant differences between wounded and control animals and credible readouts above the process control range.

Studies have demonstrated that injection of bone marrow stem cells into ischemic muscle can reduce the damage to the muscle and the loss of muscle function [17]. Bone marrow contains stem and progenitor cells which can differentiate into specific cell types such as myoblasts, chondrocytes, and endothelial cells *in vitro *and *in vivo *[41]. The role of bone-marrow-derived mesenchymal stem cells (MSCs) to directly reconstitute myoblast formation *in vivo *in damaged muscle is controversial since their main role may be that of augmenting the myogenic potential of resident muscle MSCs referred to as satellite cells [42]. *In vitro*, bone marrow cells acquire tissue-specific phenotypes when co-cultured with specialized cell types or tissue-derived extracts [41]. These potentially multipotent cells may be mobilized in the bone marrow and recruited into muscle tissue where they mitigate tissue damage following acute myonecrotic injury. Our results show that cell surface markers can be used to comprehensively track bone marrow phenotype changes associated with muscle injury in porcine compartment syndrome, which are significantly different between the control and wounded groups. Moreover, our results demonstrate that we can detect multiple putative stem and progenitor phenotypes. The large majority of these 23 phenotype subpopulations (20/23) appear to share a common minimum obligate phenotype signature (e.g. CD29+CXCR4+CD90+: Table 4), expressing markers reported to be characteristic of MSC-derived myogenic cells [25,37,43]. However, there may already be lineage-specific heterogeneity expressed by these MSC-like subpopulations in the bone marrow, since approximately half (10/23) expressed the endothelial differentiation marker CD31 [44] and an equal number (11/23) expressed the CD56 marker more commonly associated with regenerating muscle fibers and satellite cells[45]. Lineage-specific commitment can be tested by culturing such sorted MSC subsets under lineage-promoting culture conditions [41]. Based on the results presented here, the identification of bone marrow subpopulations by multiparameter FCM might be used to further sort or purify cell sets for autologous cell therapy to regenerate muscle, nerve and vascular tissues in compartment syndrome or other extremity injuries.

There are limitations to this work. First, from a biological perspective, both studies were performed with a small number of subjects. Additional experiments, including correlated memory T cell and MSC functional assays, are needed to validate the hypotheses generated by this work. Second, from an assay perspective, the analytical approach described here more readily supports those circumstances where orthogonal boundary gates (e.g. positive and negative regions) can be established. Third, from a process control perspective, the process control samples used to identify phenotypes of interest were analyzed on three consecutive days. Controls analyzed over the duration of the study would more accurately calibrate the precision of the assay. Fourth, from a computational perspective, there are practical limits to the scalability of the algorithm. Applying Exhaustive Expansion to an experiment in which there were 10 variable markers would result in a manageable 3^10 ^= 59,049 possible phenotypes, while 20 variable markers would result in a challenging 3^20 ^= 3,486,784,401 possible phenotypes.

While there is no way to alter the exponential increase in number of phenotypes as a function of the number of markers, it is unlikely that millions or billions of phenotypes would be meaningful, whether due to experimental noise (e.g. too few events to be adequately precise) or underlying biology. Thus, the phenotype search space would be pruned to a more reasonable number of phenotypes. Specific strategies for pruning the search space are beyond the scope of this work, but the general approach would mitigate the scalability impacts of the exponential increase, further extending the applicability of Exhaustive Expansion.

Furthermore, Exhaustive Expansion adds immediate value to contemporary experimental strategies and paves the way for the practical use of increasing numbers of markers. For example, one experimental design commonly published in contemporary literature uses a single fluorophore marker dump channel to exclude certain cells (e.g. CD14+, CD19+ and dead cells), two markers to identify lineage of interest (e.g. CD3 and CD4 or CD8), and another 5 markers to identify functional sets of interest (CD107a, IFN-γ, IL-2, MIP1β, and TNF-α) [31,32,46]. Using this experimental approach, 3 of the 8 total fluorophores are required to identify the parent population, while the other 5 can be considered variable identifiers of subphenotypes of interest. This construct leads to 31 sets of interest (2^5 ^- 1, since the universal set is excluded). In comparison, we have demonstrated that we can analyze over 32,000 sets, generated by 5 different panels of 8 variable markers. Additionally our approach recognizes that potential sets of interest are both those defined by all variable markers, and those defined by subsets of variable markers. Thus, our approach is readily applicable to contemporary flow cytometry experimental strategies, providing both support for an increasing number of variable markers and exhaustive interrogation of phenotypes defined by combinations of these markers.

## Conclusions

In conclusion, we have demonstrated that Exhaustive Expansion is a valuable technique for analyzing higher order polychromatic FCM data sets. Exhaustive Expansion consists of:

• generating data for all possible 0- to N-parameter sets;

• creating appropriate data visualizations;

• identifying numerically interesting sets, using such metrics as CVs and p-values; and

• inspecting the numerically interesting sets for correlative analysis of clinically or biologically meaningful results.

This approach allows us to screen hundreds to thousands of phenotypes for biological responses. Use of free, widely available, and mature software components gives us the flexibility to pursue the data analysis in directions indicated by the data itself and the associated science. Our techniques are straightforward, yet highlight intriguing results when executed exhaustively across the entire data space. They support inductive reasoning by highlighting all cell subpopulations that meet appropriate numerical criteria. In both studies discussed here, our analysis provided the foundation for a refined understanding of complex phenotypes, and allowed for the development of new hypotheses pertaining to the identification and recovery of potentially important myogenic MSC progenitor cells, and tumor antigen-specific CD8^+ ^T_CM _and T_CM _precursor populations for future clinical studies.

## Competing interests

JCS is Founder and President of CytoAnalytics. WM is Chief Technology Officer of CytoAnalytics.

## Authors' contributions

KWG and EBW designed the research. LW, DPH, and AR performed the research. JCS and WM contributed vital analytical tools. JCS, LW, AR, BZ, and EBW analyzed and interpreted the data. JCS and EBW wrote the manuscript. All authors have read and approved the final manuscript.
